# Management of chronic conditions in resource limited settings: multi stakeholders’ perception and experiences with receiving and providing integrated HIV, diabetes and hypertension services in Tanzania

**DOI:** 10.1186/s12913-023-10123-4

**Published:** 2023-10-19

**Authors:** Elizabeth H. Shayo, Jamie Murdoch, Zenais Kiwale, Max Bachmann, Mtumwa Bakari, Doris Mbata, Salma Masauni, Sokoine Kivuyo, Sayoki Mfinanga, Shabbar Jaffar, Marie-Claire Van Hout

**Affiliations:** 1 National Institute for Medical Research, Dar-Es-Salaam, Tanzania; 2 Kings College London, London, England UK; 3 University of East Anglia, Norwich, England UK; 4 Department of Statistics and Epidemiology, Muhimbili University of Health and Allied Sciences, Dar-Es-Salaam, Tanzania; 5 University College of London, London, England UK; 6 Liverpool John Moores University, Liverpool, UK

**Keywords:** HIV, Hypertension, Diabetes, Integrated care, Tanzania

## Abstract

**Background:**

The rising prevalence of non-communicable diseases (NCDs) alongside the continuing high burden of HIV poses a serious challenge to middle- and low-income countries’ healthcare systems. Pilot studies of integrated models of service delivery for HIV, hypertension and diabetes have demonstrated that they are feasible and acceptable among patients and care providers. This study assessed multi-stakeholders’ perspectives of the delivery and receipt of integrated care in Tanzania.

**Methods:**

A qualitative process evaluation was conducted in Dar es Salaam region of Tanzania where the integrated service delivery model was implemented from July to November 2021. In-depth interviews were held with seven key informants at the national, regional and district levels, eight healthcare providers, two researchers working at the integrated clinic and forty patients benefiting from integrated services at a large hospital. Three focus group discussions were held with community leaders and residents of the hospital’s catchment area, and clinic level observations were conducted. Thematic analysis was conducted followed by the use of Bronfenbrenner’s ecological model to identify factors pertinent to sustaining and scaling up of the integrated model.

**Results:**

Participants of the study at all levels were aware of the increased prevalence of NCDs specifically for hypertension and diabetes and were concerned about the trend of increasing co-morbid conditions among people living with HIV (PLHIV). The integrated service delivery model was positively perceived by stakeholders because of its multiple benefits for both patients and the healthcare system. These include stigma and discrimination reduction, improved quality of care, efficient use of limited resources, cost and time saving, reduced duplication of services and fostering of early detection for undiagnosed conditions. The organisation of the clinic was critical in increased satisfaction. Several challenges were observed, which included costs for NCD services relative to free care for HIV and inconsistent availability of NCD medications.

**Conclusion:**

Stakeholders reported numerous benefits of the integrated service delivery model that are fundamental in improving the health of many Tanzanians living with NCDs and HIV. These benefits highlight the need for policy and decision-makers to sustain and expand the integrated service delivery model as a solution to many challenges facing the health system especially at the primary care level.

## Background

Rising prevalence of non-communicable diseases (NCDs) is a major public health challenge in low- and mid-income countries (LMICs). Increasing population growth, rising life expectancy, and rapidly changing lifestyles have resulted in more NCD cases in countries already experiencing high HIV prevalence [1]. Premature deaths are common and people aged below 60 years have been substantially impacted upon by these diseases [2]. Sub-Saharan Africa has high rates of hypertension and diabetes [1], with HIV/AIDS adding to the already overstretched and poorly-resourced health systems. Many people with diabetes and hypertension live with poor quality healthcare for managing their conditions in the region, which affects their treatment continuum and related health outcomes [3]. In contrast, PLHIV in sub-Saharan Africa typically receive regular care and free healthcare services unlike those with diabetes and hypertension [4–6]. Services for HIV and other communicable diseases are generally accessible in standalone clinics with separate waiting areas, consultation rooms, pharmacies, drug procurement chains and financing. This approach often leads to the duplication of services, hence an integrated approach emerges as a potential solution as documented elsewhere [2].

Integrating NCD and HIV/AIDS services has the potential of improving health systems performance and health of individuals living with these chronic conditions. Integration of services into a single clinic model may offer a cost-effective model for treating and caring for patients with multiple morbidities [7–12]. In fact, high acceptability of integrated care services has been reported in Kenya [13], Tanzania and Uganda [14, 15]. This study assessed multi- stakeholders’ perspectives on the delivery of integrated HIV and NCD care in Tanzania to inform the scaling up of strategies, including monitoring, policy and practice development.

## Methods

### Study design and setting

The integrated care service delivery model was implemented in eight purposively selected health facilities in Tanzania [16], as a cluster randomised controlled trial with the acronym INTE-AFRICA. The selected health facilities were of medium-large sized, provided primary care and were located in largely urban and peri-urban settings of Dar es Salaam, Tanzania. Recruitment of trial participants was from 30th June 2020 to 1^st^ April 2021. In all, 3,425 patients were recruited for the trial in the intervention arm clinics where they received services in a *‘one-stop centre’,* of whom 1,710 had HIV, 246 diabetes, 781 hypertension and 688 had had either HIV and diabetes, HIV and hypertension, or all three conditions.

Healthcare provision, including setting up of the integrated care clinics within the health facilities, was carried out by health services staff, with support from the research team. Integrated clinics functioned as distinctly and separately from standard vertical care, and were predominantly held on different days. The research team organised basic training for health facility staff on the management of patients with chronic conditions, which was delivered by accredited trainers from the Ministry of Health. Where needed, the research programme contributed to the cost of the laboratory tests and supplied materials for conducting tests for the trial endpoint assessments. Training allowed a single care provider to manage HIV, hypertension and diabetes. The patients’ follow-up adhered to the routine schedule of clinic appointments, mostly every three months for stable HIV clients whereas other conditions were attended to monthly. Follow-up continued for a year after recruitment until 13th May 2022. Each off the 3,214 patients, who completed follow-up, had had at least two visits. INTE-AFRICA sought to generate evidence to inform the expansion and sustaining of chronic disease management in both Uganda and Tanzania.

This process evaluation [17] alongside the trial was conducted at one of the intervention health facilities: Mbagala Hospital in Temeke Municipality, Dar es Salaam region, Tanzania from July to November 2021. It investigated the participants’ and stakeholders’ experiences and views of integrated care, and to establish how to optimise and expand the integration of care intervention. It focused on the context of delivery, description of implementation, mechanisms of impact and outcomes, as well as the extent to which resources and activities facilitated the delivery of the intended outputs. Central to the evaluation were identifying contextually-relevant strategies for successful implementation, and practical difficulties inherent in adoption, delivery and maintenance to inform wider implementation.

#### Theoretical framework

Bronfenbrenner’s ecological model of behaviour [18] helped to conceptualise integrated care as a disruption of a complex social system [19], operating at multiple contextual levels. Our interest in the analysis was to understand opportunities for sustaining and promoting the integrated service delivery model for HIV and NCDs, which were grouped into macro, meso and micro-contextual levels (see the Table 1 below).

**Table 1:**
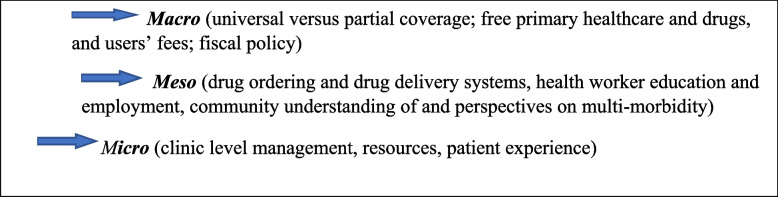
Description of three levels as per Bronfenbrenner’s ecological model of behaviour

We also investigated the contextual factors that can influence the implementation and their effects to capture variation in the adoption, delivery, and maintenance as well as the responses to the intervention.

### Study participants and recruitment procedures

Participants included patients (termed as service users) who were purposively selected to include those with either HIV, hypertension, diabetes, or a combination, enrolled into the integrated clinic at Mbagala Hospital and who had more than six months in the clinic since enrolment. The project co-ordinator and hospital clinic manager-in-charge facilitated the recruitment of patients from the database of the enrolled trial participants based on the criteria stipulated above. Other study participants were community members and leaders who were recruited from the catchment population of Mbagala hospital (Table 2). Community leaders with assistance from community health workers facilitated the recruitment of the community members including those known to have either HIV, hypertension, or diabetes. Community leaders were recruited in consultation with the hospital manager-in-charge who ensured the representation of members of the hospital’s governing body.

**Table 2:** Characteristics of the study participants

Data collection technique	Participants	Disease	Age range	Male	Female	Total
In-depth interviews	Patients	HIV	35–63	3	10	13
		Hypertension	49–78	4	4	8
		Diabetes	42–66	-	5	5
		HIV/diabetes or HIV/hypertension	54–77	4	1	5
		Diabetes and hypertension	56–77	5	4	9
	**Total**					**40**
	Care providers	**Cadres**				
		Nurses	15–55	1	2	3
		Laboratory technologist	42	1	-	1
		Pharmacist	51	1	-	1
		Clinicians	26–45	3	-	3
	**Total**					**8**
	Key informants	District level managers	39–44	2	1	3
		Regional vel managers	53	1	-	1
		National NGO representatives	-	-	3	3
	**Total**					**7**
	Clinical researchers	Focal persons in integrated care clinic	27–29	1	1	2
**Focus group discussions**	Community leaders	Ward leaders	29–38	1	1	1
		Members of health facility governing body	36–72	4	2	
	Community members	Patients	21–70	5	5	2
		Healthy individuals	29–49	4	4	
**Total participants**				**14**	**12**	**3**

Other participants were healthcare providers working in the clinics for either integrated care or NCD or Care and Treatment for HIV (CTC), key informants at the district, regional and national levels, and researchers who were the focal persons in the integrated care clinic. District informants included NCD and HIV/AIDS Control Co-ordinators and the Municipal Medical Officer. The regional informant was the HIV/AIDS Control Co-ordinator. National non-governmental organisation (NGO) representatives were drawn from the African Medical and Research Foundation (AMREF), Management and Development for Health (MDH) and Tanzania Non-communicable Diseases Alliance (TANCDA) (Table 2).

Data collection included in-depth interviews (IDIs), focus group discussions (FGDs) and one day of observation of clinical operations at the integrated clinic.

### In-depth interviews

All the 40 service users, eight healthcare providers, seven key informants and two researchers participated in participated in face-to-face IDIs. The interviews were conducted in a private place within the hospital or office environment to enhance confidentiality, privacy and audio recording. The interview guides for service users aimed to capture information on their understanding about the integrated service delivery model, its benefits and disadvantages, and practicalities of accessing services. Interviews held with clinical researchers, care providers and key informants aimed to understand their reflections on the benefits and challenges to providing and sustaining integrated services. The interviews with clinical researchers involved in the integrated care trial aimed further to illuminate on the challenges and opportunities for implementing integrated care and documenting better lessons for sustainability and scaling up nationwide.

### Focus group discussions

Three FGDs involved community members and leaders comprising males and females, including those living with one or more conditions of HIV, hypertension, or diabetes. For community members, males and females had separate discussions to enhance freedom of expression while community leaders’ discussion combined both males and females. Eighteen community members and eight community leaders participated in the group discussions (Table 2). The FGDs conducted in the community setting at the street government offices also aimed to document the contextual factors related to delivery to and receipt of integrated care services for service users with HIV, hypertension and diabetes, including challenges to and opportunities for sustainability.

All the interviews and FGDs were conducted by experienced social scientists, who had received prior orientation on the study objectives, ethics, methods and data collection tools. They were moderated using Kiswahili, Tanzania’s national language, and audio-recorded after obtaining informed consent from the study participants. The IDIs lasted between 45 and 60 minutes whereas FGDs took about 90–120 minutes.

### Observations

Observations were conducted at the integrated care clinic targeting specific areas where service users interacted with healthcare providers. The observer focused on the overall physical structure of the hospital and location of the integrated clinic and on specific service points (registration, clinician room, laboratory and pharmacy). Observations also focused on seating arrangements in waiting areas, discussion among service users while waiting for services, movement of service users from one service point to another, and health education talks. Observations were carried out by a senior social scientist, with handwritten field notes taken.

### Data management and analysis

Data were collected and analysed by three experienced social scientists, who had received training on how to conduct the qualitative study. They were informed about the study objectives, data collection techniques, and ethical regulations and conduct. Interview and FGD audio-recordings were transcribed verbatim and later translated into English. The transcripts were then checked for accuracy using back translation. The research team read and re-read several transcripts before developing codes. Double coding was done for the initial transcripts and emerging codes were presented and compared amongst the investigators for consensus before embarking on coding of the remaining transcripts.

A Microsoft Excel sheet facilitated the categorisation of the findings in relevant codes and nodes. Thematic analysis was used to interpret responses among and between levels of informants, working iteratively between different data sources to strengthen interpretations. Bronfenbrenner’s ecological model’s ‘macro’, ‘meso’ and ‘micro’ contexts [18] were used to categorise contextual influences on delivery of integrated care at the point of implementation, to enable us to generate concrete recommendations for sustaining and scaling up the integrated model. The analysis also focused on identifying strengths, weaknesses, opportunities, and threats to integrated service. Triangulation of observational, interview and FGD data was conducted to test and extend emerging interpretations. This paper has adhered to reporting guidelines for qualitative research [20].

### Ethical considerations

Ethical clearance and oversight were provided by the research ethics committees of the Liverpool School of Tropical Medicine in the United Kingdom, and the National Institute for Medical Research in Tanzania. Local government authorities and health facilities accordingly granted permission to conduct the evaluation. All the study participants provided written informed consent for their voluntary participation in the study. We assured the participants of confidentiality, privacy, and anonymity throughout the evaluation.

## Results

### Demographic characteristics

A total of 40 service users were interviewed, of whom 24 were females and 16 males aged 35 -78 years, and 17 had health insurance (See Table 2). All the participants had more than six months exposure to integrated care (ranging from 7–12 months) and nine continued receiving integrated care after completing the 12 months follow-up period. Sixteen out of 40 participants engaged in small businesses whereas 15 did not report engaging in any income generating activities. The remaining participants were employed in the private sector or were engaged in farming activities. The age range for the eight healthcare providers was 24–55 years, with their working experience ranging from 6—15 years. Among the 18 FGD participants for community members, 10 were living with chronic disease conditions (six of whom had HIV, two diabetes, two a combination of these disease conditions), and the rest were healthy individuals. Among the eight community leaders who participated in the FGDs, five were members of the health facility governing body (Table 2). All the key informants occupied managerial roles and had a biomedical background.

The findings fall under four themes: i. Stakeholders experiences on HIV, diabetes and hypertension; ii. Benefits of integrated care service delivery; iii. Challenges of integrated care service delivery; and iv. Sustaining and scaling up of integrated care.

### Stakeholders’ experiences on HIV, diabetes and hypertension

The main focus was on their awareness of the three conditions, the perceived trend, and the risk factors contributing to the trend observed.

All the study participants revealed their awareness of the increasing prevalence of chronic diseases including HIV and NCDs, especially hypertension and diabetes. For example, a female nurse said that of the 80—90 patients they attend to each day, 60 would be found with hypertension. This burden was mainly attributable to changing lifestyles, eating habits, lack of physical exercise, personal behaviour and poverty:

*“The chronic diseases, which we have witnessed, apart from HIV, are hypertension and diabetes. Currently, many people have hypertension; it is a leading disease even though you may find that patients with hypertension may also include those with HIV or diabetes. The main cause is living Western type of life, we eat everything, no exercise…”* (Key informant - regional level)

### Benefits of integrated care service delivery

Service users, care providers and key informants were asked to reflect on the integrated service delivery model. Their views on the benefits on integrated care are reported below under subheadings corresponding to the emerging sub-themes and associated codes (Table 3).

**Table 3:** Sub-theme and codes on the benefits of integrated care

Sub-Theme	Code categories
Integration enabling patients to live their lives	Positive attitude towards receiving service in the same clinic Good quality of services Satisfaction with health education provided Reduced disruptions in care seeking Time and cost saving Increased knowledge Continuity of care; medicines availability; follow up support; Reduced complications
Integration minimising stigma and discrimination	Good organization of the clinic that enhances privacy Location of the clinic outside CTC clinic Patients shared multiple services hence difficult to be identified Equity in service delivery Indoor medicine dispensing
Integration as a solution to late detection and monitoring of disease conditions	Close monitoring of patients; comprehensive screening in each visit; availability of competent providers Increased knowledge of patients with three conditions Availability of blood pressure and blood glucose machine
Integrated service delivery model as a solution to poorly resourced health systems	Cost saving to patients with multiple conditions; cost saving in diagnostic services at the health facilities Prevent double use of diagnostic kits for a single test/screening Effective human resource utilization A single care provider managing the three conditions at a time
Integrated services and efficiency in time management	Shorter waiting time for patients Time saving to care providers Timely disease management

#### Integration enabling patients to live their lives

Service users were positive about receiving care for different conditions in the same clinic on the same day. Moreover, they demonstrated an understanding of the importance of ongoing management with regular medication. Furthermore, they appreciated the quality of services and health education provided on dieting and medication adherence. Healthcare providers, clinical researchers, community members, programme co-ordinators and managers also hailed the integrated service delivery model for reducing disruptions in the patients’ everyday lives, saving time and money, particularly for those seeking recursive permission from work to attend multiple clinics in different locations:

*“It is a very nice thing, indeed. If it was to be made sustainable, it could help a lot.... You find one patient attending three different clinics because of being HIV-positive, hypertensive, and diabetic. So, the patients attend a diabetic clinic at Temeke, a hypertension clinic at Bunju, HIV clinic at Mbagala. Integration of these services in one place minimises disturbance”* (Healthcare Provider, male).

Regional and district stakeholders reported that before the introduction of integrated healthcare service, complications to patients increased when these diseases (i.e., HIV, hypertension, and diabetes) were managed separately. For example, viral loads among children with HIV increased because of non-adherence. Weight gains due to antiretroviral intake coupled with poor dietary education were perceived to contribute to other NCDs:

“*…*This *is a challenge to us service providers because to reduce someone’s weight means that person must refrain from eating certain foods; on the other hand, we advise this person to eat dietary and nutritious food so that his/her can gain enough immunity; therefore, you find yourselves jumping into second challenge, the big burden is hypertension and diabetes”* (Key informant - District, level).

Nevertheless, the integrated service delivery model was appreciated for its potential in clearing misconceptions through improved health education.

Healthcare providers also reported increased knowledge among clients with different disease conditions and their associated risk factors that facilitated proper management, follow-up, and counselling of patients. District stakeholders reported that patients were reluctant to return to their original vertical clinic because of the benefits accruing from attending the integrated clinic: *“I came twice to this integrated clinic and saw how grateful patients were- i.e.. … for coming and receiving care they had expected to get…”* (Key informant-district level).

#### Integration minimising HIV related stigma and discrimination

Healthcare providers and service users reported how integrated care was delivered without discrimination:

*“Integration significantly minimises discrimination because all the patients at the waiting area do not know one another’s condition until they enter the doctor’s room… We do discuss [with service users ] on few things so it helps to reduce the discrimination”* (IDI, in-charge of care and treatment centre, Male)*.*

Another participant said:

*“You know in other facilities our clinic is so open that everyone will know that person is HIV-positive. But combining with hypertension and diabetes makes it difficult for one to know your problem. This has made it very easier, when I was told that I have completed my schedule so I will be sent back there [to CTC], I was very shocked”* (Male patient, 56 years old, HIV).

**Fig. 1:**
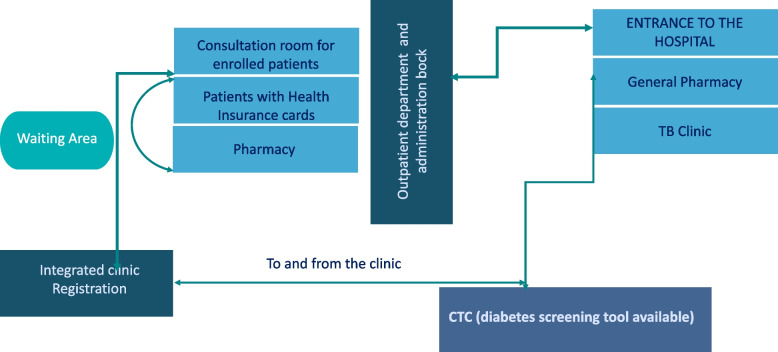
Patients flow

The way the clinic was organised was critical in enabling patients to progress with ease from one service point to another— from registration, consultation, to the medicine dispensing room, which was also confirmed through observations (see Fig. 1).

#### Legend: Arrows indicate patients’ pathway to different sections after entering the health facility.

Observations revealed limited space at the health facility under study. In consequence, the hospital management innovatively located the integrated clinic behind the administration block instead of the ambulatory CTC to enhance privacy. In addition, dispensing medicines indoors made it difficult for outsiders to distinguish patients with different conditions, including HIV/AIDS. Similar findings were reported by community members:

*“I think integration will eliminate discrimination and the number of patients will increase even for those who feared to collect their medicines and be seen or exposed. Everyone will collect his/her medicines and leave”* (FGD-Community Members, Females).

Nevertheless, care providers and clinical researchers reported that the small waiting areas, as well as patients attending the clinic at different times, made it difficult to deliver joint health education. As a result, clinicians provided one-to-one education in consultation rooms, which gave patients freedom to ask any questions. Health education reportedly motivated patients to continue attending integrated care even after completing the scheduled 12-month follow-up. Overall, most participants said that the location of an integrated service was an important variable in boosting its acceptability and recommended providing a ‘neutral’ setting at the Mbagala hospital:

*“… most of the time transferring HIV patients from their department to another one might pose a challenge. Again, if this service is available at the HIV clinic, the diabetes and hypertension clients may not be that happy to go there because the general population knows that the clinic is for those with HIV. To simplify it, we can organise integrated clinic in different locations”* (Key informant-NGO representative).

Regional informants, healthcare providers, NGO and community representatives perceived the integrated service delivery model as a stigma reduction strategy for patients, which allowed PLHIV to continue receiving care normally: “… *if we meet at the service point will you ask why have you come here? We have all come to seek the same services”* (Key informant– regional level). Implicitly, the integrated service delivery had changed the dynamics of healthcare provision.

Study participants emphasised that the value of integration for identifying different conditions was clear, particularly for PLHIV. However, key informants at the district level cautioned that patients with different conditions might avoid mixing with others when receiving services, especially if the integrated clinic was located in the building which had previously provided exclusive HIV care:

*“Aah people with HIV harbour the feeling called stigma. This has been a problem, which makes patients not to mix with others, but also what I found is that some patients with HIV have NCD challenges in terms of diabetes and pressure …* (key informant- district level).

All HIV clients at the integrated clinic felt free to attend and receive services there, but a few reported residing far from the hospital and, thus, travelled to avoid community stigmatisation. This stigmatisation of HIV was reflected by NCD clients, who were enrolled in the integrated clinic and lived in the vicinity. They initially feared to enter the HIV clinic building for diabetes screening. Fearing neighbours would think they had HIV, they opted to attend a general laboratory for diabetes testing. Eventually, however, they came to accept integrated service delivery over time.

#### Integration as a solution to late detection and monitoring of disease conditions

Healthcare providers and key informants including NGO representatives appreciated the integrated service delivery model as strategic in fostering early detection of other disease conditions due to close monitoring of the patients and comprehensive screening during each visit. They claimed that non-integrated services left many people living with undiagnosed conditions until their disease had progressed and reached a critical point, particularly diabetes and hypertension, which are known as silent killers because many clients tend to be unaware they had these problems. This view was supported by clinical researchers who reported finding clients enrolled into integrated care with hypertension but unaware of this condition:

*“Yes, some people end up with strokes unknowingly. But s/he had hypertension for a long time without knowing. The time a person gets stroke, while the mouth deviates to the right or left may be due to hypertension; but if would get this education early enough in this integrated clinic, I think cases of strokes wouldn’t be many…”* (IDI, Health Care Provider-Pharmacist, Male)

Healthcare providers and key informants argued that living with undiagnosed conditions resulted from community members having limited knowledge on the signs and symptoms, lack of culture of undergoing screening for multiple conditions, unavailability of screening tools and untrained staff at lower-level health facilities, poor client-provider relationships and services unaffordability. In some instances, better-off individuals, suspecting they had hypertension or diabetes, could buy over-the-counter drugs from pharmacies without consulting health facilities. It was observed that these challenges were overcome through integrated care clinics because of the comprehensive health education, availability of screening tools and trained healthcare providers, continued monitoring of all the conditions through screening during each visit, and encouraging patients to have health insurance cover.

The integrated clinic had diagnostic tools such as blood pressure and blood glucose machines. One district-level manager described how such patients were monitored before integration was introduced:

*“…before integration, what I found was that some HIV patients had NCD challenges in terms of diabetes and hypertension. But when they came to the HIV clinic, they were rarely screened for blood pressure and sugar levels.”* (Key informant-district level).

Some of the study participants complained about inconsistent medicines supply as a barrier to sustaining integrated care. In this regard, healthcare providers said that, before the introduction of integrated service, complaints about medicine availability were linked to the health system’s arrangements that made prescriptions and dispensing dependent on the level of healthcare facilities (whether at the hospital, health centre or dispensary level).

#### Integrated service delivery model as a solution to poorly-resourced health systems

Human resources and cost-saving were mentioned as advantages of the integrated model, benefitting both the health system and individual patients. Due to the shortage of human resources experienced in healthcare facilities, district key informants perceived integrated service delivery as an efficient approach to maximising the use of the staff available in treating multiple conditions at a single service point:

*“I would recommend continuing with this model as it is very helpful, not only to the client as an individual but also to the health facility. In other words, it is possible to use modest resources for big achievements; it is just to send one staff member for training in managing multiple disease conditions. By doing so, you will have saved human resources and automatically you serve the client [much more effectively], hence creating more [client] satisfaction…”* (Key informant-district level)*.*

The integrated care model was perceived as a cost-saving for patients, who had an added advantage of saving the costs of diagnostic services. Previously, a single patient would be subjected to double charges for the same medical investigation offered in different clinics:

*“…this patient can today come to me for NCD, which is hypertension, and you find that I want to know the functioning of his kidney, liver, and so on but the same patient two weeks ago came for HIV services and required to do similar tests. It adds up a lot of costs”* (Key informant- district level).

#### Integrated services and efficient time management

Informants perceived that integrated care one saved time in moving from one service point to another. Comprehensive laboratories investigations were perceived to save time for the technicians that could otherwise be spent on performing similar tests requested by different clinicians for the same co-morbid patients at different times. Significantly, patients receiving integrated services reported shorter waiting times unlike before receiving integrated care:

*“I am thankful to these interventions that have continued to integrate services in one place like HIV, diabetes, hypertension. It means someone coming and receive intensive services at the same time i.e., the patient comes one day and gets all the services and leaves. Then, we hope he will continue with his/her economic activities faster and his health will improve further”* (Key informant - district level)

The regional HIV co-ordinator also reported shorter waiting time benefits, reflecting on the situation before integration was introduced:

*“Someone may be allocated to two clinics on the same day and would have to queue up for both services; for example, they will queue up for HIV, and then will come to queue up again for hypertension. The long waiting time really poses a big challenge in healthcare...”* (Key informant-regional level).

Efficient time management was also observed in the delivery of investigation results, especially for patients with co-morbidity, which fostered informative and comprehensive clinical decisions contrary to what used to happen before healthcare integration. Previously, diagnosis and disease management were done in peace-meal manner at the discretion of the clinicians.

### Challenges of the delivery of integrated care services

#### NCD service costs and structural challenges

In contrast to HIV services, patients enrolled in the integrated clinic had to pay for NCD services, including consultation and medications, although they received blood pressure and sugar level screening free-of-charge from the clinic. This constituted a barrier to effective execution of the integrated care model. Even though the exemption policy for the very poor and elderly exists, community leaders complained about its ineffective implementation since some patients could not get all services they needed for free. This was confirmed by healthcare providers who reported prescribing drugs to NCD patients to purchase from a pharmacy, which was perceived as disadvantage for low incomes patients and without health insurance. As a result, patients with NCDs were encouraged to enrol into health insurance schemes to ensure uninterrupted and unhindered access to the health services they needed:

*“We see absolutely the survival of people with HIV and retention is stable. Clients come for medicine refill but for those with hypertension and diabetes who do not have insurance it is a challenge and, sometimes, you find that their survival is declining. If you observe it in terms of integration a big percentage of clients is for those with hypertension (around 40 percent)”* (IDI, Care Provider, Male).

Healthcare providers and district informants reported that without health insurance, however, costs would continue to be a barrier to the utilisation of integrated services for hypertension and diabetes.

Additionally, at the initial stages of implementing the integrated care, longer waiting time were experienced by some enrolled diabetes patients, who decided to receive screening services at the general laboratory with no preferential treatment for patients with diabetes and hypertension. As time progressed, they continued receiving these services at the CTC where the screening tool was placed.

#### Availability of trained healthcare providers

Regional and district managers, and healthcare providers argued that the current level of training of healthcare providers, typically undergraduate or diploma level, was too insufficient for them to manage and treat different conditions in an integrated clinic. In addition, staff turnover undermined efforts to provide optimal integrated care and its sustainability:

“*…Staff have been transferred now and again; you train him/her today and you find him/her transferred tomorrow, and they are sent to facilities which do not provide such [integrated] services*” (Key informant- district level*).*

### Opportunities for sustaining and scaling up the integrated service delivery model

#### Supportive environment

Patients appreciated the humility and friendliness of integrated care service providers and mentioned some names for the kindness, politeness, and good language they used when providing health education and instructions on medicine adherence. This finding was supported by the continuing attendance of the service users (9/40) even after 12 months of receiving integrated care had elapsed. The CTC in-charge of one of the facilities confirmed the existence of these good relationships.

Furthermore, the availability of health facilities and clinics at different levels throughout the country emerged as an opportunity for scaling up the integrated care service delivery:

*Of course, clinics are available everywhere/in all places; it is for us to use them for integration but ensure drugs are available, people are trained, etc, [otherwise] everything is there. There are partners who do provide support, so it is for us to play our role” ***(**Key informant-regional level).

However, the informants emphasised the importance of ensuring privacy and confidentiality to prevent stigmatisation, to patients with different conditions.

#### Structural arrangements

A series of supportive supervisory sessions, provided by leaders and partners at the national, regional and district levels from the routine services and projects management was perceived by healthcare providers as an asset in sustaining the integrated care delivery. The supervision was reported to be supportive in addition to enhancing discussions on the challenges they experienced, what worked and did not work, to find solutions together. The presence of NCD and HIV co-ordinators in each municipality and health facility (hospital or health centre) enhanced the prospect of scaling up the integrated care service delivery model. Health facilities had focal persons to organise and manage clinic activities with support from administration unit:

“*We usually plan together the entire budget to avoid duplication of activities. At the district level we have the NCD co-ordinator specifically for NCD activities…We must have assurance of utility when we decide to introduce integrated services, such as human resources and facilities to have equipment and drugs. This will ensure continuity of such services* (Key informant- district level).

The health facilities were further empowered by the council to perform their duties and prepare budget based on the needs of clients.* “…Health managers have to include issues of NCD screening equipment and medicines when developing the Comprehensive Council Health Plans. Let them include those needs in the plan so that they can be funded”* (Key informant-NGO representative)*.*

These practices emerged as better avenues for sustaining and scaling up the integrated service delivery model. However, the multiple aspects of the management of NCD such as varieties of drugs and equipment complicated the budget plan and modality to ensure universal access of care for patients. Key informants at the district and regional levels who were NCD and HIV coordinators, and NGOs representatives said that the health system must have adequate resources when deciding to scale up and sustain the integrated services. Sufficient human resources were argued as critical, as well as facilities with the appropriate equipment, and a good supply of medicines.

#### Support from the implementing partners

District and regional managers acknowledged the support from the NGOs and other development partners in the delivery of different health services such as HIV, NCDs and tuberculosis. These partners were seen as the avenue to advocate for integrated care in the respective regions/districts:

*“Yah, we are collaborating with stakeholders. They do provide training and employ staff. We have staff that MDH has hired to support care and treatment for HIV. The support has gone up to the community by training community health workers. MDH is a big stakeholder. We have over a hundred staff employed by MDH and are paid through them”* (Key informant, district level).

Sensitisation of the local community on the importance of attending to the integrated clinics was reported by community leaders. Key informants at the regional level reiterated the importance of developing evidence-based guidelines on integrating care services to avoid external political influence.

 The presence of the guidelines stipulating how, where, what and who will implement integrated care would formalise the process of scaling up and sustaining the model. Equity to people in accessing integrated healthcare services, which was mentioned as critical, include decentralisation to primary care levels, promoting insurance coverage, and effective exemption mechanisms for the very poor. Table 4 provides a list of additional aspects that all the key informants and care providers mentioned would foster scale up and sustainability of integrated care.

**Table 4:** Facilitators for sustainability and scaling up of integrated care as per the ecological theory

Macro	Meso	Micro
• Development of guidelines and SOPs on service integration and distribute them to practitioners • Enforcing the exemption policy • Employment of staff to the facility with shortage • Funding considerations to also cover NCD services for free as it is for HIV	• Infrastructural development to ensure privacy • Service point to be neutral and well accessible • Availability of medicine and screening tools • Availability of trained personnel who know the chronic disease burden • Improvement in interpersonal trust Equity in service provision • Provision of health education and educational materials on integrated care • Good provide-client relationship • provision of NCD services free of charge as it is for HIV	• Accessibility of the services • Awareness about the integration • Affordability of transport and services • Friendly and health facility environment • Enhance a culture of health check and timely care seeking • Change in attitude and misconception about diseases

## Discussion

The process evaluation revealed a range of positive reflections on the integrated service delivery model in Tanzania from patients, care providers, community members, researchers, district and regional co-ordinators and NGO representatives. Study participants generally favoured the integrated model of care as it facilitates early detection of disease conditions, saves cost and time for patients and health systems generally, fosters timely and comprehensive management of patients, enhances interpersonal relationships, and improves patients’ health due to continual multi-morbid health education and follow-ups. The model is perceived as a stigma reducing strategy. Indeed, locating the integrated clinic outside the building in which HIV-only care was provided has been seen as one the factors for increased acceptability. This development also demonstrates the potential of integrated care helping to address the heightened trend of NCDs alongside high HIV prevalence that has doubled the disease burden in poor and malfunctioning health systems [2, 21].

In the experience of the Tanzanian health systems, the benefits achieved in the integrated clinic can be difficult to attain in non-integrated clinics, especially in government health facilities. Currently, there are restrictions imposed on providing specialised services at lower levels of care such as dispensaries and health centres, including the types of medicines available at these service points. Moreover, in the government health systems specialists can only be allocated to district, referral, and national hospitals. This requirement does not apply to private health facilities, where care providers from different levels and cadres are frequently employed. The challenge emerges that even though lower-level private facilities might employ specialists in managing multiple NCDs, the national health insurance (NHIF) policy limits prescription at given levels. As a result, clients accessing private primary care facilities with a national health insurance card lack access to certain medications and, thus, pay out-of-pocket [22]. Introducing integrated services at the primary care could also alleviate such concerns as it would go hand-in-hand with removing these barriers from the NHIF operations.

The Tanzania government has waiver mechanisms for the very poor and the elderly including those with NCDs to access care through the exemption policy. However, implementation is problematic as politicians often advocate for free access to services for such groups when, in practice, they will need to pay for medicines and diagnostic tests. Similarly, other studies have found that elderly individuals with hypertension and diabetes, who lack access to screening services and receiving incomplete dosages of prescriptions, and therefore instructed to buy the remainder from pharmacies [23]. Help Age International has called on all stakeholders to ensure that coverage and quality of health services meet the needs of all the elderly in relation NCDs and enhance non-discriminatory practice to prevent individuals and their family from languishing in abject poverty due to prohibitive healthcare costs [24]. Other studies have also reported limited and irregular supplies of screening equipment, medications, lack of adequate training of healthcare workers on managing NCD, and treatment costs and payment systems as hurdles to effective integration of care services [25].

Overall, the integrated care service delivery model remains an avenue for delivering health education covering the three disease conditions, fostering behavioural change in dieting, engendering medicine adherence, and facilitating early detection of other disease conditions. After all, insufficient knowledge of different conditions and associated risk factors has contributed to the persistent rising trend for NCDs with many people living with these conditions undiagnosed [26]. For example, insufficient knowledge among clients with HIV attending the CTC can compromise their health when other conditions emerge [25]. Increase in weight for PLHIV highlights how integration is important for monitoring these clients. Our findings indicate that, with integrated service delivery, there are reduced complications for clients due to increased knowledge among healthcare providers in managing a combination of diseases and associated risk factors. Also, inadequate trained human resources, medicines, and diagnostic tools are significant barriers to achieving universal health coverage which raise critical ethical questions when introducing integrated care model [27].

Facilities implementing the integrated service delivery model were reported to have improved the quality of care in terms of waiting time, trained personnel, dispensing of drugs, availability of screening tools, and enhanced interpersonal trust. This model can also reduce stigma while bolstering non-discrimination since all clients, regardless of their disease conditions, get fair treatment without prejudice. Against this backdrop, it is evident that running of disease-specific clinics is a waste of resources. Integration can enhance efficient use of scant resources available while easing cases of co-morbidities.

Similarly, stakeholders have also foreseen challenges for clients with co-morbidities as they visit multiple clinics, implying more time and costs spent on care. PLHIV contend with high risk NCD-related factors that threaten their survival [21, 28], yet they receive care from standalone clinics, which inevitably affect their treatment continuum and related health outcomes [3]. Similar observations emerged among HIV clients attending the CTC whose referrals to NCD clinics were initiated after NCD conditions emerged [25]. The increased HIV-NCD burden of diseases in Tanzania, as in other sub-Saharan African countries, demands more efforts aimed to integrate healthcare services and enhance universal health coverage.

Clients with single and multiple conditions who received services from the integrated clinic have indicated greater acceptance which was also supported by NGOs implementing HIV and NCD activities, regional and district stakeholders, and community leaders and members. The model was thought to be cost-effective, with risk aversion benefits, and without endangering care quality and expected outcomes for HIV clients [12, 29]. These benefits also emerged during the review of evidence from Sub-Saharan Africa [2] and, therefore, integrated model appears to be feasible, effective, efficient and acceptable [14, 15]. This study can inform government actions to expand the integrated model to the rest of the country.

### Strengths and limitation

This study provides original views about integrated care from a wide range of stakeholders across the health system including patients, care providers, researchers, district, regional and national stakeholders and community leaders and members. Their views are substantial enough to inform sustainability and scalability plans. Different data collection techniques were employed, and data were triangulated to enhance validity and trustworthiness of the findings. Service users interviewed had different durations of exposure to the interventions offering a breadth of experiences over time. Interviews for service users who continued attending the integrated clinic after completing the 12 months of follow-up reinforced the strengths of implementing this integrated model due to the increased trust and confidence in services delivered. However, service users and care providers recruited in this evaluation study were limited to only one hospital setting, making it difficult to generalise findings to other health facilities in different regions.

## Conclusion

An integrated service delivery model could improve the health of many people living with NCDs and HIV in Tanzania. Improved service quality and efficient use of resources, interpersonal relationships and health education were important indicators for increased acceptability. Although conflicting findings have emerged on the cost for NCD relative to the free services for HIV clients and NCD medicine availability, enrolled clients continued receiving care in the integrated clinic. Such positivity supports the case for scaling up and sustaining of the integrated healthcare delivery model. Nevertheless, guidelines development on integrated service delivery and ensuring universal access to care for NCD and HIV clients are important prerequisites for scaling up the integrated model.

## Data Availability

The datasets used and/or analysed during the current study are available from the corresponding author on reasonable request.

## Abbreviations

CTC: Care and Treatment Centre
HIV/AIDS: Human Immunodeficiency Virus infection and Acquired Immune Deficiency Syndrome
LMICs: Low- and Mid-Income Countries
NCD: Non-Communicable Diseases
NHIF: National Health Insurance Fund
PLHIV: Patient Leaving with HIV
SSA: Sub Saharan Africa
